# A cross-cultural study showing deficits in gaze-language coordination during rapid automatized naming among individuals with ASD

**DOI:** 10.1038/s41598-021-91911-y

**Published:** 2021-06-28

**Authors:** Kritika Nayar, Xin Kang, Jiayin Xing, Peter C. Gordon, Patrick C. M. Wong, Molly Losh

**Affiliations:** 1 Roxelyn and Richard Pepper Department of Communication Sciences and Disorders, Northwestern University, 2240 Campus Drive, Frances Searle Building, #2-366, Evanston, IL 60208 USA; 2 Department of Linguistics and Modern Languages, The Chinese University of Hong Kong, Shatin, Hong Kong SAR China; 3 Brain and Mind Institute, The Chinese University of Hong Kong, Shatin, Hong Kong SAR China; 4 Psychology and Neuroscience, University of North Carolina at Chapel Hill, Chapel Hill, NC USA

**Keywords:** Autism spectrum disorders, Biomarkers, Cognitive neuroscience, Language

## Abstract

Individuals with autism spectrum disorder (ASD) and their first-degree relatives demonstrate automaticity deficits reflected in reduced eye-voice coordination during rapid automatized naming (RAN), suggesting that RAN deficits may be a genetically meaningful marker of ASD language-related impairments. This study investigated whether RAN deficits in ASD extend to a language typologically distinct from English. Participants included 23 Cantonese-speaking individuals with ASD and 39 controls from Hong Kong (HK), and age- and IQ-comparable groups of previously-studied English-speaking individuals with ASD (n = 45) and controls (n = 44) from the US. Participants completed RAN on an eye tracker. Analyses examined naming time, error rate, measures of eye movement reflecting language automaticity, including eye-voice span (EVS; location of eyes versus the named item) and refixations. The HK-ASD group exhibited longer naming times and more refixations than HK-Controls, in a pattern similar to that observed in the US-ASD group. Cultural effects revealed that both HK groups showed longer EVS and more fixations than US groups. Naming time and refixation differences may be ASD-specific impairments spanning cultures/languages, whereas EVS and fixation frequency may be more variably impacted. A potential underlying mechanism of visual “stickiness” may be contributing to this breakdown in language automaticity in ASD.

## Introduction

Rapid automatized naming (RAN) involves serial naming of arrays composed of common symbols (numbers, letters) and non-symbols (colors, objects), with the objective of completing the task as quickly and accurately as possible. This simplicity of the RAN task belies the complexity of the different component skills underlying RAN, including core executive (e.g., working memory, processing speed, inhibitory control of attention)^1^, and linguistic skills (e.g., retrieval of lexical information, phonological processing and memory)^2^, as well as the ability to coordinate these processes. RAN is commonly used as an index of automaticity for reading, and has been identified as one of the strongest predictors of successful reading across multiple languages^3^. An extensive network of underlying neural correlates have been implicated in RAN ability (e.g., frontal cortex, temporo-parietal areas, dorsal posterior regions, inferior frontal cortex, and the ventral-visual pathway and cerebellum)^4–7^, which highly overlaps with the language-reading network. Not surprisingly, then, RAN has been studied as a measure of language fluency that taps basic executive and language skills underpinning higher-level language skills, such as reading^3^ and narrative^8^. Moreover, impairments in RAN performance have been documented in many language-related disorders, including autism spectrum disorder (ASD)^8–10^. Given that reading deficits are not typically observed in ASD (with subgroups of individuals actually exhibiting outstanding word reading ability^11,12^, repeated observations of RAN deficits in ASD appear to reflect differences in skills tapped by RAN but not specific to reading. Similar RAN-related differences have also been documented among unaffected relatives of individuals with ASD^8,10^, who too show subtle differences in complex communication (e.g., social language)^13–16^. For example, early family studies of individuals with ASD have shown links between literacy and language-based skills in their siblings and parents^17^. A recent study similarly revealed protracted development and lower performance in early literacy and language-based skills during childhood among *parents* who then went on to have a child (or children) with ASD as adults^18^. Twin studies have also demonstrated that RAN abilities are highly heritable^19^, highlighting RAN as a neurobiological marker of genetically influenced language and executive abilities^20–24^, that might be fruitfully studied to understand the basis of language-related impairments across different neurodevelopmental disorders.

Inefficient RAN performance, specifically, longer naming time and greater error rates, has been documented in individuals with ASD relative to typical controls^8–10^. RAN naming was also found to be associated with social communication skills and repetitive behaviors (RRBs) in ASD^8,9^, suggesting that the skills tapped by RAN performance (naming and errors) may importantly relate to clinical symptoms of ASD, including global executive and social-communicative impairments. Slower naming and greater error rates have also been observed among clinically unaffected first-degree relatives (both siblings and parents) of individuals with ASD^8–10^, pointing towards RAN as a marker of language-related mechanisms possibly influenced by ASD genetic liability.

Studies examining eye fixation patterns during RAN have begun to delineate more specific breakdowns along the processing stages involved in RAN, among both individuals with ASD and their first-degree relatives. For instance, eye voice span (EVS; the number of items ahead the eyes are in comparison to the voice at the onset of each vocal response) is thought to index the fluent coordination of pre-processing abilities (visual input in working memory) and the conversion of this visual input to phonological code and articulatory commands^3,25,26^, encompassing the influences of basic language processes (e.g., syntactic and semantic processing)^27^ and working memory^28^. Fixation frequency and the type of fixation (e.g., perseverations and regressions) also index important skills underlying language processing fluency, namely, local processing/attention. For example, regressions, or, the backward movement of the eyes to prior items during RAN, can indicate a failure to plan and organize in advance, resulting in need for self-corrections^29–32^. On the other hand, a greater number of perseverations is an indication of a perseverative or local attentional style (i.e., “sticky attention”)^26,30^. Prior work reported shorter EVS and a greater number of fixations and refixations (perseverations and regressions) among individuals with ASD and their first degree relatives^8,9^, providing an important indication of key mechanistic disruptions during RAN processing that can inform the language-related impairments in ASD, and that are likely influenced by ASD genetic risk. Additionally, perseverative and regressive eye movement patterns have been shown to relate to poorer narrative quality and increased sensorimotor behaviors in those with ASD^8^, suggesting a connection to ASD-related clinical symptoms. Together, findings demonstrate how careful examination of eye movement and speech during RAN might reveal inefficiencies or impairments in the basic mechanisms that provide the foundation for more complex, downstream language abilities such as narrative skill.

Previous studies exploring RAN performance in ASD have been restricted to English-speaking individuals in the United States (US)^8–10^, leaving unclear whether RAN differences may be present in speakers of languages that are typologically distinct from English language and culture, and whether RAN deficits represent a robust marker of impacted skills underlying the ASD language phenotype. For example, prior work in English- and Italian-speaking children with specific language impairments, showed that Italian speakers had greater control of phonological aspects of speech than did English speakers, highlighting the impact of phoneme-grapheme mapping differences that are more inherent to some languages than others^33^. Similarly, a review paper highlighted phonological processing and awareness differences in languages that differed in orthographical transparency (e.g., Greek/German versus English/French)^34^. Differences in linguistic features across languages can also result in deficits that are specific to speakers of those languages. For instance, Cantonese-speaking children with specific language impairment^35^ and Mandarin-speaking children with ASD^36^ display impairments in lexical tone perception that could not manifest in the same way in non-tone languages. In a more direct cross-language comparison, English-speaking children with dyslexia are known to have marked deficits in phonological awareness^37^ but morphological, rather than phonological awareness is a primary feature of dyslexia of Chinese^38,39^. Cross-linguistic comparisons are therefore critical in understanding potential language-specific profiles in ASD and other neurodevelopmental disorders, particularly across languages that are typologically distinct, and potentially imposing unique structural challenges.

Prior studies exploring RAN performance in typical development and dyslexia have demonstrated strongly consistent findings across languages. For instance, in both typically developing Chinese and English speakers, quicker RAN naming and longer EVS were found in symbolic (letters and digits) versus non-symbolic (colors and objects) conditions^40–42^. Additionally, children with dyslexia in both East Asian and Western cultures were shown to demonstrate similar patterns of differences from their respective control groups, with slower RAN naming and shorter EVS, and more extensive impairment emerging in the symbolic conditions^42–44^. RAN has also been widely studied across languages as a longitudinal and concurrent predictor of reading ability (particularly for predicting reading accuracy and reading fluency)^45,46^, with RAN-reading ability correlations of similar strength reported across languages that differ in orthography (e.g., English, Greek, Chinese)^45^.

Several studies have now demonstrated impairments in aspects of RAN performance—from slower naming to decreased eye-voice coordination and efficiency during naming—in ASD and among clinically unaffected relatives who are at increased genetic liability^8–10^, as well as in groups who carry a high confidence ASD risk gene^47^. An important next step in understanding the role of these language-related impairments in ASD risk is to document RAN performance in languages other than English. Although prior work has not investigated RAN in Chinese, a language that is inherently distinct from English, eye-tracking studies conducted in East Asian populations have tapped into similar deficits in RAN-related skills in ASD, with mixed evidence for a linguistic or cultural effect. For example, similar to patterns demonstrated in ASD groups from Western cultures, Chinese children with ASD demonstrated a preference of looking at repetitive movements versus random movements in a preferential looking paradigm^48^, which may derive from slower attentional disengagement or “sticky” or local attention^49,50^. Additionally, impaired cognitive flexibility in Chinese children with ASD was also found and was reflected in their visual scanning patterns ^51^. In contrast, some key differences in eye-movement patterns have emerged across East Asian and Western cultures in the general population, implicating an effect of culture. For instance, findings from Lee et al. (2016)^33^ revealed a wider distribution of fixations (i.e., global processing/attention) across scenes depicting faces in Taiwanese participants, compared to more centrally-focused fixations (i.e., local processing/attention) in American participants.

This study builds on these prior observations of RAN impairments in ASD and first-degree relatives in Western populations, by investigating RAN performance and gaze patterns in children and adults with ASD in an East Asian culture (i.e., Hong Kong; HK). Previously published data from English-speaking individuals with ASD and controls were included for comparison. We predicted that individuals with ASD from HK would show similar RAN performance and fixation patterns as the US ASD group^8–10^, and show slower naming speed, greater error rates, shorter EVS, and higher fixation and refixation frequencies than their age-matched controls. Finally, we also predicted that impaired RAN performance would correlate with greater ASD symptom severity. As noted, data from the US sample were reported previously^8^ and were included for comparison with new data obtained from HK participants, in order to examine potential cross-cultural differences in RAN. Data from the US sample have not been re-analyzed independently, nor have they been re-produced here as standalone findings.

## Methods

### Participants

Participants included 23 individuals with ASD and 39 controls from Hong Kong (HK) and age- and IQ-comparable samples from the US (n = 45 individuals with ASD and 44 controls) who have been previously studied^8^. All participants from HK spoke Cantonese as their first language, as well as English. Participants from the US spoke English as their primary language. Every effort was made to ensure that samples were comparable, while also ensuring maximum sample sizes. Specifically, a minimum age of 10 years was set to mitigate the impact of developing rapid naming automaticity^53^, and age and IQ were included as covariates for most statistical analyses (see below). Participants from HK were recruited using advertisements on social media platforms (e.g., Facebook) and flyers that were distributed to schools and organizations. Participants from the US, who were included in a prior publication^8^, were recruited through study advertisements distributed to ASD clinics and advocacy organizations, participant registries, and word of mouth.

All participants were screened for family history of genetic disorders related to ASD (e.g., fragile X syndrome), dyslexia, or brain injury (based on parent report). Additionally, all participants had normal or corrected-to-normal visual acuity, IQ ≥ 80, and chronological age ≥ 10 years (see Table 1 for sample characteristics). ASD diagnostic status was confirmed using the Autism Diagnostic Observation Schedule-Second Edition (ADOS-2)^54^ modules 3 or 4 and/or the Autism Diagnostic Interview-Revised (ADI-R)^55^ for US participants. For the HK-ASD group, ASD status was reliant upon participant’s report, and/or report from the clinics/schools from where the participants were recruited. HK-ASD participants also received the ADOS-2 modules 3 or 4 upon study enrollment to examine ASD symptomatology as a correlate. Of the HK-ASD participants, 15 participants met criteria for ASD based on the ADOS. For quality control purposes, exploratory analyses were conducted to compare RAN performance between those HK-ASD participants who met ADOS ASD criteria and those who did not. Findings did not differ between groups (*p*s > 0.081), suggesting that overall findings were not likely driven by one HK-ASD subgroup versus the other.

**Table 1:** Sample characteristics—RAN.

	Hong Kong		United States		Culture effect		Diagnosis effect		Diagnosis X culture interaction	
	Control group	ASD group	Control group	ASD group						
	M (SD) [range]	M (SD) [range]	M (SD) [range]	M (SD) [range]	F	*p*	F	*p*	F	*p*
n (M/F)*	39 (20:19)	23 (18:5)	44 (21:23)	45 (36:9)	–					
Age (years)	20.5 (6.2)	17.9 (8.9)	19.5 (5.4)	17.9 (6.1)	0.24	0.627	3.84	0.052	0.18	0.671
	[10.2–31.9]	[10.1–32.9]	[10.5–33.3]	[10.0–35.1]						
PIQ	110.8 (13.5)	106.4 (11.8)	112.9 (14.4)	104.1 (14.9)	0.006	0.936	**9.42**	**0.003**	0.83	0.364
	[82–139]	[84–126]	[79–143]	[68–131]						
ADOS total severity score^^^	–	4.9 (2.5)	–	6.6 (2.3)	0.04	0.848	–﻿			
		[1–10]		[1–10]						

Bold indicates significance *p* < .05.

*Sample size reflects image with maximum number of participants.

^^^ADOS total comparison severity score labels are as follows: 0–2 = “minimal-to-no evidence”, 3–4 = “low”, 5–7 = “moderate”, 8–10 = “high”. ADOS modules administered across samples included modules 3 and 4.

IQ was measured using the non-verbally administered Test for Nonverbal Intelligence—Fourth Edition (TONI-4) for HK participants and Wechsler Intelligence Scale for Children—Third Edition (WISC-III) or the Wechsler Abbreviated Scale of Intelligence (WASI) for US participants. The nonverbal standard score derived from the TONI-4 has been shown to be highly correlated with the Perceptual Reasoning Index from the WISC-III^56^, and the instruments are comparable in their measured constructs. As such, nonverbal IQ from the TONI-4 for HK participants and Performance IQ (PIQ) from the Wechsler scales for US participants were considered to represent “IQ” henceforth.

### RAN procedures

Participants completed a RAN task (both non-symbolic or colors/objects and symbolic or numbers/letters) from the Comprehensive Test of Phonological Processing (CTOPP)^57^. The four stimulus types included two runs each and procedures were identical to Nayar et al. (2018)^8^. In short, each run contained an array of 36 items, and participants were instructed to name as accurately and rapidly as possible from left to right in each row, and from top to bottom for all rows. Before each stimulus type, a practice row of nine symbols was completed by all participants to ensure comprehension of task instructions. Color runs depicted colored circles and object runs depicted simple pictures of objects that participants were required to label. Participants’ gaze was tracked during RAN using a Tobii TX300 (sampled at 60 HZ) series eye tracker for HK participants and a Tobii T60 series eye tracker for US participants. Participants were seated approximately 50–60 cm from the screen. Gaze was calibrated prior to the task using a standard 5-point grid built in Tobii Studio. Participants were re-calibrated following any large movements; accuracy of calibration was 0.5°. An eye-tracking/calibration window was monitored for calibration deviance during task administration. Finally, calibration checks were embedded in the task (e.g., center cross-hair in between runs) to ensure tracking accuracy across runs. Stimuli were presented on a 21’’ TFT monitor in HK and a 17’’ TFT monitor in the US, with a resolution of 1280 × 1024 pixels. Digit and number runs were displayed in black on a gray background, while color and object runs were colored on a gray background. Voice responses were recorded using an external USB microphone.

Participants were tested in a quiet laboratory space or at the participant’s residence, with comparable administration procedures and environments across groups. All study procedures were approved by respective University Institutional Review Boards (Northwestern University and The Chinese University of Hong Kong) and all methods were performed in accordance with the relevant guidelines and regulations. Written informed consents and assents when applicable were obtained from all participants and/or their parent/legal guardian for study participation.

### Data processing

RAN variables of interest (see below) were averaged across both runs for each stimuli type. They were then further averaged into non-symbolic (colors, objects) and symbolic (numbers, letters) conditions, which were used in final analyses. Research has previously demonstrated significant differences between symbolic and non-symbolic performance in RAN, such that symbolic conditions are thought to be more automatically processed after 6 years of age^8,44,58^, and have been shown to be more impaired in ASD^8,9^.

#### Vocal responses

HK participants completed most conditions (non-symbolic conditions and the number runs) in Cantonese. Although English was their second language, and because Cantonese is a non-alphanumeric system with no direct translation of the English alphabet to the Cantonese language, HK participants completed the letter runs in English. It is also noteworthy that English language instruction is the standard in schools in HK, starting as early as 2 years, 8 months. As such, HK participants had many years of practice with the English alphabet system, and so these stimuli were highly familiar to both groups of participants, permitting stronger experimental control than using different stimuli across cultures. Importantly, there were no significant differences in RAN naming time between letter and number runs within HK participants (condition effect; F(1, 52) = 0.12, *p* = 0.74) and group X condition effect; F(1, 52) = 0.00, *p* = 0.98)), indicating comparability between the two symbolic conditions.

The onset and offset of the articulation of each item was marked using the Penn Phonetics Lab Forced Aligner, an automatic and forced phonetic alignment toolkit that synchronizes phonetic transcriptions with speech signals^59^. The onset and offset boundaries were further examined by trained coders who were blind to participant diagnosis, marking errors or deviations reflective of incorrect naming^8,9,60^. Based on the beginning and ending time of each trial, eye movement and vocal responses were aligned, following methods outlined in Nayar et al. (2018)^8^.

#### Gaze

For each RAN item, the AOI was defined as a region extending vertically and horizontally from the center of each item to the midpoint between adjacent items see for details ^8,9^. Fixations were assigned to an AOI based on their spatial coordinates, and consecutive fixations within the same AOI were pooled and counted as ‘perseveration’. Quality control procedures followed prior work^8^, and included the following steps: (1) Parameters before exporting gaze data were set according to published standards^61^, to account for potential data loss in working with neurodevelopmental populations. These included usage of the built-in I-VT fixation filter in Tobii Studio set to a strict average across both eyes, a velocity threshold of 35°/s, 100 ms duration gap and 0.5° between each new fixation; (2) a minimum fixation duration for unique fixations was set at 100 ms; (3) runs with track loss of > 35% of total fixation duration were excluded; (4) runs with no fixation duration for > 10 s were excluded; (5) consistent with prior work^8,60^, eye movements associated with the first two and last four items of each array were excluded from analyses (due to increased eye-tracking variability and subsequent challenges inherent to interpreting such eye movements, including mistargeting and long saccades back to the beginning of a row); and (6) minimum and maximum number of fixations per run were established based on outliers (> 2.5 SD above mean) and data distributions. For all samples, this was set at 15 and 50 for letter/number trials, and 20 and 55 for color/object trials, respectively.

Overall, 11%, 1%, 7% and 12% of individual runs were excluded for the HK-control, US-control, HK-ASD, and US-ASD groups, respectively. 2X2 chi-squared revealed significant differences in the proportion of trials excluded by group (*Χ*^2^ (2, *N* = 1400) = 9.1, *p* < 0.01); the control group from the US demonstrated significantly fewer excluded runs compared to the other three groups. The total number of runs included in the final analyses, however, did not differ significantly by group (*Χ*^2^ (2, *N* = 1208) = 2.0, *p* = 0.16).

#### RAN naming performance variables

*Naming time* was calculated as the time used to articulate all 36 items on each run, including errors.

*Frequency of errors* was calculated by summing all errors and self-corrections during naming, including repetitions, omissions, or substitutions.

#### RAN eye movement variables

*Eye-voice span (EVS*) was defined as the lead in the eyes compared to the item being spoken. EVS parameters were identical to those reported in Nayar et al. (2018)^8^.

*Number of fixations* was defined as the total number of fixations regardless of type (see below) made during the run.

*Refixations* were defined to capture the type of fixation occurring. These included perseverations (the number of consecutively repeated fixations within the same item) and regressions (the number of times there were backward eye movements towards previously-visited items) that occurred during the run.

### Statistical analyses

Data were examined to determine appropriate statistical models (parametric versus non-parametric), revealing normal distributions (via visual inspection of histograms and Q–Q plots due to relatively small sample sizes) across conditions, cultures, and diagnoses for all but one variable. Unsurprisingly, errors rates were positively skewed, primarily due to their low occurrence. As such, analyses involving error rates were re-analyzed using a series of Mann–Whitney U non-parametric tests.

For all variables, a series of linear mixed effects regression models were conducted separately for the symbolic and non-symbolic conditions using the lmer package^62^ for R^63^. Fixed effects included diagnosis (ASD versus TD), culture (HK versus US), and their interaction term (culture X diagnosis interaction), with sex, and age added as control variables. IQ was also included as a covariate for RAN naming performance variables (naming time and errors), which is consistent with previous studies^8^. Random effects included the random-by-participant intercept. Model comparisons were conducted between models with and without the interaction term to determine model fit. Stepwise model comparisons revealed no significant differences between models, and as such, all subsequent analyses included the interaction term to address the current study’s primary goals. P-values for linear mixed effects models were adjusted using the Benjamini–Hochberg method^64^ using a false discovery rate (FDR) of 0.10. FDR level of 0.10 was selected to adjust for multiple tests without potentially missing important effects^65^ due to relatively smaller sample sizes. Least square means (LSM) post-hoc pairwise comparisons were additionally performed for models showing a significant main effect or interaction effect using the lsmeans package^66^ (*p*-values adjusted using Tukey adjustments). As noted previously, the US-ASD and US-control participants were all included in prior work^8^, and are reported here to provide a basis for comparison with HK groups. Readers are referred to this prior study for pairwise comparisons examining effects between US groups, which are not repeated here. Finally, partial Pearson correlations with age and/or IQ covariates were conducted to assess how RAN performance variables may relate to ASD symptom severity in individuals with ASD based on the overall comparison score derived from the ADOS. Associations were only examined in the HK-ASD group, and not re-examined in the US-ASD group.

### Ethical approval

All procedures performed in this study were in accordance with the ethical standards of the institutional research committee and with the 1964 Helsinki declaration and its later amendments or comparable ethical standards.

### Informed consent

Informed consent was obtained from all individual participants included in the study.

## Results

**Figure 1:**
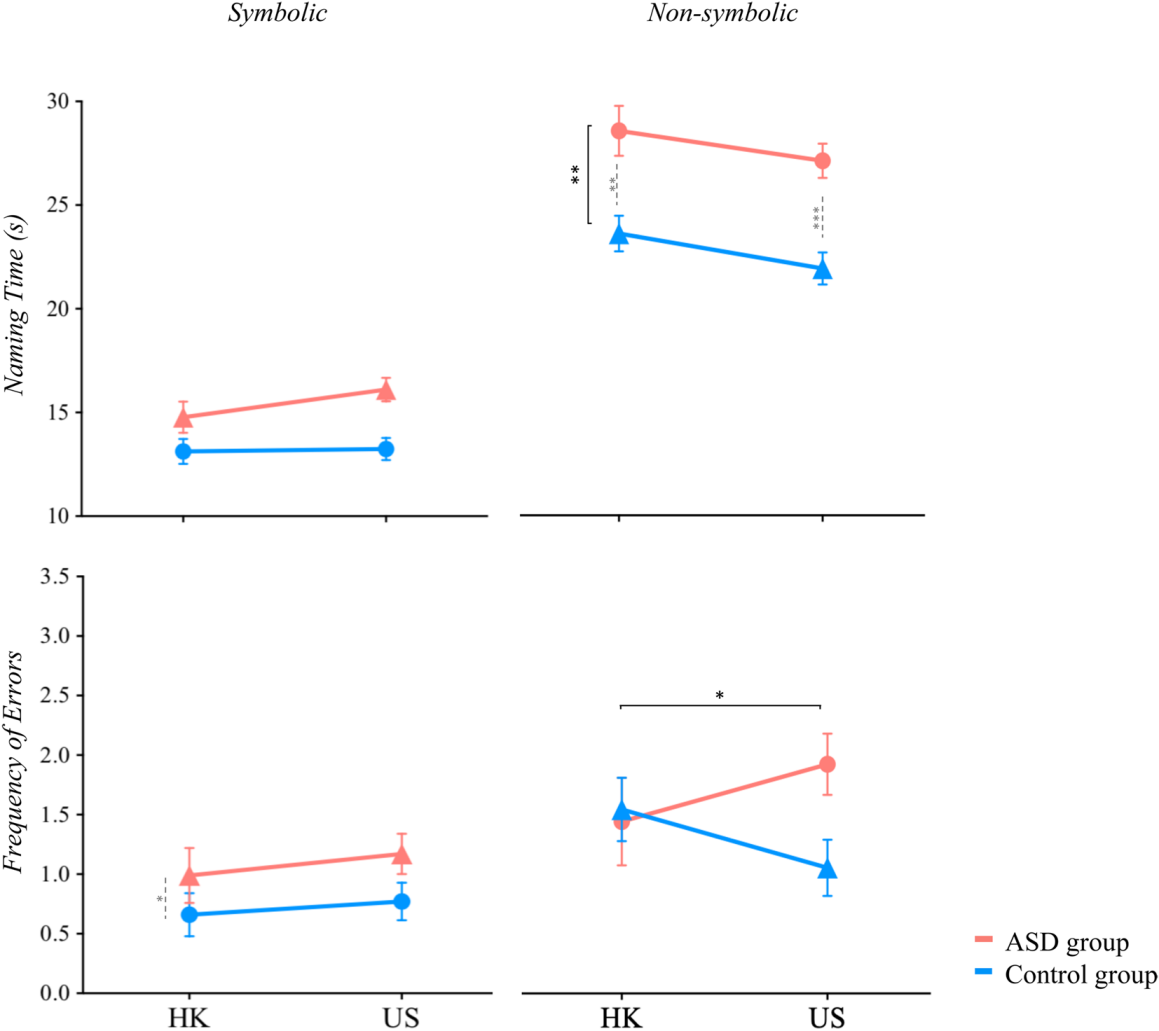
RAN performance (naming time and error rates) across cultures, diagnostic groups, and conditions. A significant diagnosis effect emerged in the non-symbolic condition for naming time (*Estimate* = − 4.80 *p* < .01). Error bars represent standard error of the mean (SEM). For US-ASD versus US-control group comparisons, refer to Nayar et. al., 2018^8^. *p < .05, **p < .01, ***p < .001. Black overall bars denote significance of the overall model (i.e., group effect, diagnosis effect), and dashed grey lines indicate pair-wise comparisons.

**Figure 2:**
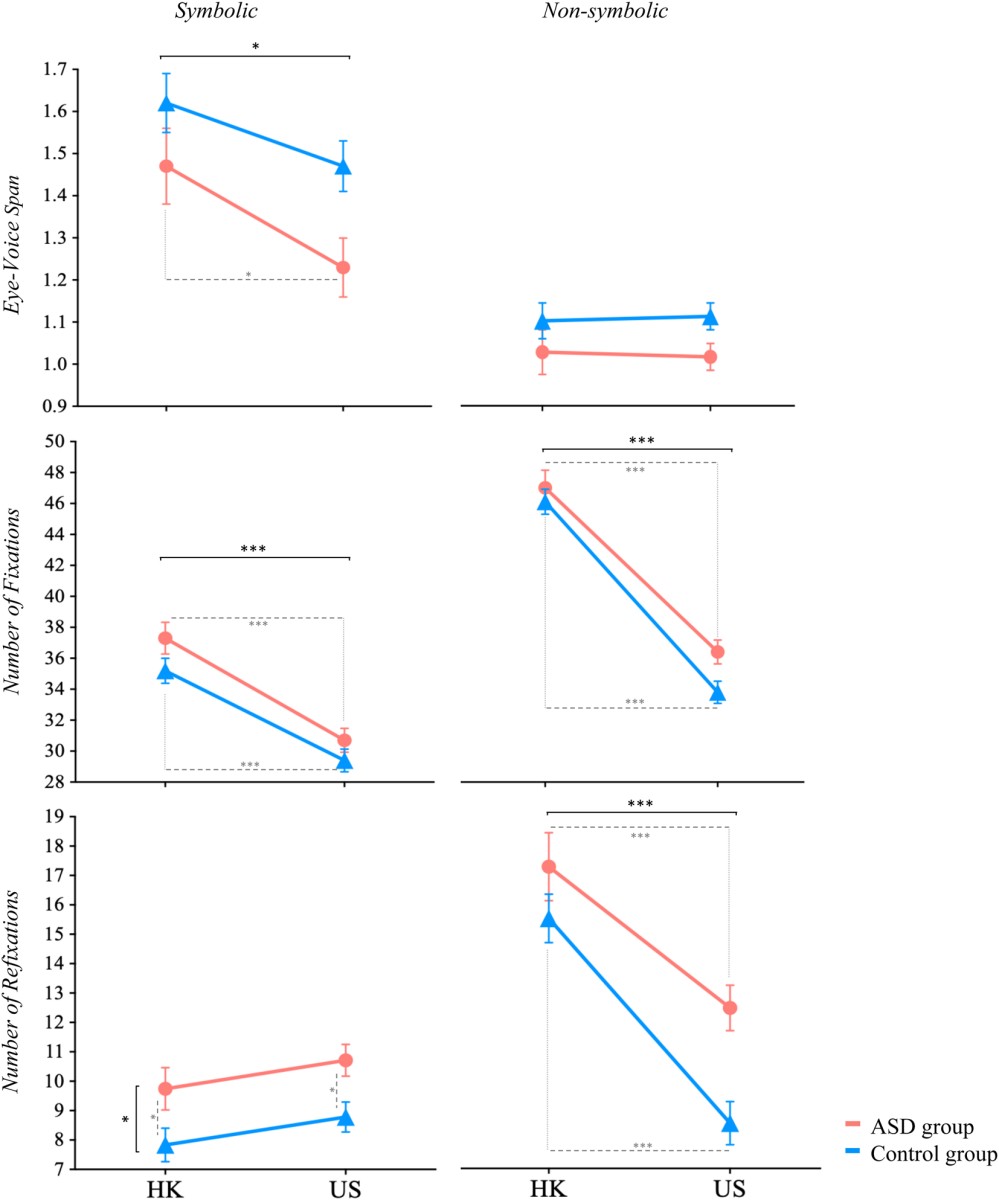
RAN eye-movement variables across cultures, diagnostic groups, and conditions. Culture effect for EVS symbolic condition (*Estimate* = − 0.23, *p* < .05) and total fixations across conditions (Estimates < -6.52, *p*s < .0001). Diagnostic effect for refixations during symbolic condition (*Estimate* = −0.190, *p* < .05) and culture effect during non-symbolic condition (*Estimate* =− 4.77, *p* < .001). Error bars represent standard error of the mean (SEM). For US-ASD versus US-control group comparisons, refer to Nayar et. al., 2018^8^. *p < .05, **p < .01, ***p < .001. Black overall bars denote significance of the overall model (i.e., group effect, diagnosis effect), and dashed grey lines indicate pair-wise comparisons.

Descriptive statistics and group statistical comparisons are presented in Table 2 and Fig. 1 (Naming performance) and Fig. 2 (Eye movement during RAN), and summarized below.

**Table 2:** Summary of results.

Variable	Condition	Hong Kong		United States		Culture effect					Diagnosis effect					Diagnosis X culture interaction				
		Control	ASD	Control	ASD															
		M (SE)	M (SE)	M (SE)	M (SE)	*Est*	SE	*t*	*p*	*padj*	*Est*	SE	*t*	*p*	*padj*	*Est*	SE	*t*	*p*	*padj*
**Performance**																				
Naming time (s)	Symbolic	13.13 (0.60)	14.77 (0.76)	13.24 (0.54)	16.11 (0.57)	1.34	0.90	1.50	0.137	0.274	−1.64	0.97	−1.68	0.095	0.317	−1.24	1.20	−1.03	0.305	0.763
	Non-symbolic	23.18 (0.84)	27.98 (1.17)	21.54 (0.75)	26.57 (0.80)	−1.41	1.37	−1.03	0.304	0.380	−**4.80**	**1.44**	−**3.33**	**0.001**	**0.010**	−0.23	1.77	−0.13	0.896	0.984
Errors	Symbolic	0.66 (0.18)	0.99 (0.23)	0.77 (0.16)	1.17 (0.17)	0.18	0.27	0.66	0.509	0.566	−0.33	0.30	−1.10	0.273	0.341	−0.07	0.37	−0.20	0.846	0.984
	Non-symbolic	1.51 (0.26)	1.41 (0.36)	1.03 (0.23)	1.88 (0.25)	0.47	0.43	1.11	0.270	0.380	0.11	0.45	0.24	0.812	0.812	−0.96	0.55	−1.74	0.084	0.763
**Eye tracking**																				
Eye-voice span	Symbolic	1.62 (0.07)	1.47 (0.09)	1.47 (0.06)	1.23 (0.07)	−**0.24**	**0.11**	−**2.18**	**0.031**	**0.077**	0.16	0.12	1.37	0.174	0.341	0.08	0.14	0.55	0.582	0.984
	Non-symbolic	1.09 (0.04)	1.02 (0.05)	1.10 (0.03)	1.01 (0.03)	−0.01	0.06	−0.09	0.927	0.927	0.07	0.06	1.14	0.256	0.341	0.02	0.08	0.22	0.825	0.984
Total fixations	Symbolic	35.20 (0.82)	37.30 (1.04)	29.40 (0.74)	30.70 (0.78)	−**6.52**	**1.24**	−**5.25**	** < .0001**	** < .0001**	−2.03	1.34	−1.51	0.133	0.332	0.65	1.66	0.39	0.698	0.984
	Non-symbolic	46.1 (0.81)	47.0 (1.13)	33.8 (0.72)	36.4 (0.77)	−**10.53**	**1.32**	−**7.95**	** < .0001**	** < .0001**	−0.80	1.40	−0.57	0.567	0.630	−1.86	1.72	−1.08	0.280	0.763
Refixations	Symbolic	7.84 (0.57)	9.74 (0.72)	8.79 (0.51)	10.71 (0.54)	0.97	0.86	1.13	0.260	0.380	−**1.90**	**0.92**	−**2.06**	**0.042**	0.208	−0.02	1.14	−0.02	0.984	0.984
	Non-symbolic	15.46 (0.82)	17.21 (1.15)	8.54 (0.73)	12.44 (0.78)	−**4.77**	**1.34**	−**3.56**	**0.001**	**0.002**	−1.75	1.42	−1.24	0.218	0.341	−2.15	1.74	−1.24	0.218	0.763

Bold indicates significance *p* < .05; padj included Benjamini–Hochberg correction at a false discovery rate of .10.

### Naming performance

#### Naming time

There were no significant culture, diagnosis, or culture X diagnosis effects during the symbolic conditions. For non-symbolic conditions, a significant diagnosis effect emerged (*Estimate* = − 4.80, *p* = 0.001), such that individuals with ASD across both cultures were significantly slower than their respective control groups (HK: *t*(134) = 3.33, *p* = 0.001; US: *t*(134) = 4.51, *p* < 0.0001). There was no significant effect of culture or culture X diagnosis interaction for the non-symbolic conditions.

#### Frequency of errors

There were no significant culture, diagnosis, or culture X diagnosis effects in error rates across both symbolic and non-symbolic conditions. Mann–Whitney U tests revealed significant differences in error rates during symbolic trials for HK groups, showing that the HK-ASD group committed a greater number of errors than their respective HK control group, (*U* = 4.89, *p* = 0.027). Additionally, the HK control group committed a greater number of errors than the US control group during non-symbolic trials (*U* = 5.86, *p* = 0.015).

### Eye tracking performance

#### Eye-voice span (EVS)

During the symbolic condition, there was a significant culture effect (*Estimate* = − 0.23, *p* = 0.031)—individuals with ASD from HK had a significantly longer EVS than individuals with ASD from the US (*t*(141) = 2.18, *p* = 0.031). No diagnosis or culture X diagnosis interaction emerged for the symbolic conditions. Similarly, there were no significant culture or diagnosis effects or interactions during the non-symbolic conditions.

#### Number of fixations

Across both conditions, a significant culture effect (symbolic: *Estimate* = − 6.52, *p* < 0.0001 and non-symbolic: *Estimate* = − 10.53, *p* < 0.0001) was detected, with both groups from HK making a significantly greater number of fixations compared to their US counterparts (symbolic: ASD *t*(141) = 5.25, *p* < 0.0001, TD *t*(141) = 5.35, *p* < 0.0001; non-symbolic: ASD *t*(135) = 7.95, *p* < 0.0001, TD *t*(135) = 11.45, *p* < 0.0001). No significant diagnosis or culture X diagnosis interactions emerged in either condition.

#### Refixations

During the symbolic condition, a significant diagnosis effect (*Estimate* = − 1.90, *p* = 0.042) revealed that across both cultures, individuals with ASD made a greater number of regressions and perseverations than their respective control groups (HK: *t*(141) = 2.06, *p* = 0.042, US: *t*(141) = 2.53, *p* = 0.012). No significant culture or culture X diagnosis interaction emerged for the symbolic condition. During the non-symbolic condition, a significant culture effect emerged (*Estimate* = − 4.77, *p* = 0.001), where both groups from HK made a greater number of regressions and perseverations than their US counterparts (ASD: *t*(135) = 3.56, *p* = 0.001, TD: *t*(135) = 6.32, *p* < 0.0001). No significant diagnosis or culture X diagnosis interaction emerged during the non-symbolic conditions.

### Clinical-behavioral correlates of RAN ability in individuals with ASD from HK

No significant associations between any RAN performance or gaze variables with ADOS scores emerged for individuals with ASD from HK.

## Discussion

This study explored the coordination of gaze and language during rapid automatized naming (RAN) in individuals with ASD and controls from both Hong Kong and the US, to examine whether RAN impairments previously documented in ASD might extend beyond linguistic and cultural boundaries, and constitute a marker of language-related impairments associated with ASD. Specifically, prior work studying English-speaking individuals with ASD and their relatives reported key differences in rapid naming across both performance (i.e., longer naming time) and gaze (i.e., longer eye-voice span, elevated total fixations, and increased rates of refixations)^8–10^. The HK-ASD group showed some similarities with this profile, but an apparent cultural effect was observed in some areas. Specifically, the HK-ASD group did not differ from controls in eye voice span (EVS) or in their overall fixation rate, and both the ASD and control HK groups differed from the US groups on these component skills, suggesting a cultural effect likely stemming from visual processing and attentional cultural differences. In contrast, as was previously documented in US-ASD groups, the HK-ASD group demonstrated longer naming times and elevated rates of refixations compared to controls. It is thus possible that this specific pattern of naming and gaze during RAN may be tied to ASD-risk genes, particularly as they have been documented in ASD across cultures, among first-degree relatives of ASD^8,9^, as well as among carriers of the *FMR1* premutation (a gene heavily implicated in ASD)^8,47^.

Diagnostic effects detected for naming time in the non-symbolic conditions across both HK and US groups are consistent with prior findings from US samples, demonstrating longer naming time among individuals with ASD^8,9^, as well as their siblings^9^ and parents^8,10^. That no cultural or culture by diagnosis effects emerged during these non-symbolic trials (or during symbolic conditions), where participants from HK spoke in their native language, is consistent with prior work demonstrating similar RAN naming in control groups between cultures e.g., ^40,42,67^. More importantly, group differences in the absence of such culture effects suggest that RAN naming time in non-symbolic trials may be more sensitive to ASD-related deficits across cultures than the symbolic (i.e., letters and numbers) condition, perhaps reflecting the increased cognitive demands inherent to non-symbolic than symbolic conditions (Norton & Wolf, 2012). Norton and Wolf (2012)^3^ also highlighted the role of cognitive processes involved in lower-level functions (e.g., visual processing, phonological representation) to be accurate and automatic. When the lower-level functions are not operating efficiently, this may have downstream effects on the allocation of resources to multiple high-order language skills such as reading, conversation, and narrative. Indeed, a recent meta-analysis documented relationships between RAN symbolic conditions and reading speed (a lower level process) in contrast to relationships between RAN non-symbolic conditions and reading comprehension (a more advance reading skill)^68^. Prior work has also indicated that the stream from perceptual encoding to articulatory processes in RAN is more susceptible to interference during non-symbolic conditions^60^, with greater challenges suggesting reduced automaticity due to inefficient linguistic and executive processes. Given the overlap in reading and language networks and brain regions implicated in RAN, it is then not surprising that individuals with a language-related disorder such as ASD, and those with a greater genetic liability to ASD (siblings/parents) will have greater difficulty during non-symbolic conditions than their control counterparts, regardless of culture or language.

Findings that the control and ASD groups from HK showed comparable naming time during the symbolic trials contrast with prior work from the US^8,9^, and may be attributable to the requirement that Cantonese-speaking participants name letters in English (for stimuli consistency). Both groups took approximately two seconds longer to name letters than numbers, and this lag may have dampened possible diagnostic effects. In terms of naming error rates, while non-parametric findings revealed a greater number of errors in the HK-ASD group compared to HK controls (and reported for US group in Nayar et al. (2018)^8^, and in HK control compared to US control groups, there were very few errors overall (~ 1–2 errors on average), reducing interpretability owing to this variable’s lack of sensitivity e.g., ^8,9^.

Findings from eye movement analyses, which highly complement the performance-based measures of time and errors by reflecting underlying strategies or mechanisms, revealed more complex differences across groups and cultures, and arguably provided a more sensitive index of RAN-related deficits than global measures of naming and error performance. Specifically, culture effects were found in EVS, wherein EVS was longer in both HK groups compared to those from the US, particularly during the symbolic condition. Moreover, though not statistically significant, EVS in both the ASD groups was shorter than their respective controls, indicating comparable diagnostic group patterns across English- and Cantonese-speaking individuals with ASD. It is possible that systematic education that starts from as early as 2 years and 8 months in HK, may have conditioned children in HK to pay closer attention to symbolic information. In addition to indexing automaticity and coordinating visual-vocal processing, EVS may also tap global visual processing, known to be different across Eastern and Western cultures. For instance, the lead in eye movement may signify a more seamless and efficient ability to process visual information more rapidly, and therefore, globally^69–71^—perhaps processing information further along in the stimulus more rapidly. According to Hofstede’s ‘individualism–collectivism framework’^72,73^, Western individualistic cultures (e.g., U.S.) value independence and self-expression, whereas Eastern collectivist cultures (e.g., Japan, China) emphasize social connection and harmony with the environment. Additionally, consistent with observations that Eastern and Western cultures differentially emphasize collectivism versus individualism, evidence from studies of visual perception suggest that members of East Asian cultures have demonstrated a greater propensity toward processing global information (background or the “gist”) versus Western cultures^52,74^. It is therefore possible that global processing in ASD is only somewhat dampened in East Asian cultures, and therefore protective against an ASD effect, as compared to individuals with ASD in Western cultures who have the tendency to process information more locally^75^. This may have resulted in an EVS that was comparable to that of the US control groups’, but longer (and therefore more efficient overall; as reflected by relationships with faster naming across groups and cultures; data not shown given prior work consistently evidencing this relationship)^8,9,26,27,60,67^ than that of the US-ASD group. Finally, differences in EVS during symbolic conditions between ASD groups across cultures may have risen due to methodological differences in naming languages, although there were no culture or diagnosis differences in the non-symbolic conditions, indicating their efficacy in tapping into skills that are not influenced by culture or ASD^8^. As such, differences between the HK and US groups are unlikely due to language differences, particularly as the words in each task are largely mono-syllabic.

Findings related to fixations may have arisen from methodological differences that placed an unequal task burden in Cantonese than in English. Specifically, both groups from HK tended to make a greater number of fixations compared to both groups from the US, and the number of refixations differed by group and culture, depending on the condition. There were cultural effects in the non-symbolic condition where both groups from HK refixated more often than both groups from the US. Increased refixation rates during this non-symbolic condition appeared to also prolong naming time for both HK groups, and error rates for the HK-control group, which may be related to methodological differences in language used for naming. While for English, initial sounds in items displayed in the object and color trials were phonetically unique (e.g., pen, star, fish, chair, boat, key), there were a greater number of overlapping initial-phonemes in Cantonese (e.g., bat1, seng1, jyu4, dang3, syun4, so2 si4). Given, prior work evidencing the co-activation between phonological codes of phonologically similar words and fixations during reading, which prolonged reading times^76–78^, it is possible that similarities in initial-phoneme sounds may have therefore impacted the ability for HK groups to efficiently and automatically convert the visual information into phonetic information, potentially influencing both naming time and fixation style.

During symbolic trials, during which both US and HK participants labeled letters in English, a diagnosis effect emerged, such that individuals with ASD across both cultures committed elevated rates of refixations than their control counterparts. Furthermore, in line with the local–global visual processing hypothesis, both groups from the US made a greater number of refixations (i.e., greater local processing) than both groups from HK, with both ASD groups demonstrating this visual rigidity or “stickiness” more prominently compared to their control counterparts. Importantly, this fixation pattern may reflect ASD genetic influence as it robustly differentiated groups in two independent studies of RAN and eye-voice patterns conducted with first-degree relatives of individuals with ASD, as well as among carriers of the *FMR1* gene in its premutation (a gene heavily implicated in ASD)^8,47^.

Finally, the lack of associations with ASD symptomatology in the current study is not necessarily surprising, given the relatively limited battery of clinical-behavioral measures available, consisting of a single overall symptom severity score derived from a clinical measure (ADOS) that was normed on individuals from Western cultures. Prior work has demonstrated relationships with more detailed information about social-communication traits and RRBs from spontaneous narrations of story-books and conversational interaction in parents of individuals with ASD^8^, suggesting that it will be important for future work to examine relationships between RAN and a broader range of phenotypic measures, including those that are normed in East Asian cultures, that may more sensitively capture more nuanced ASD-related atypicalities.

## Conclusions and future directions

Taken together, differences detected in ASD across cultures in naming time and refixations in particular have implications for understanding the language phenotype of ASD. Links with language-related phenotypes have been previously identified with evidence showing reduced synchronization of voice and eye movement during RAN were related to narrative skills in ASD^8^. Additionally, findings that similar differences in RAN have been documented among populations with subtle pragmatic (social) language atypicalities^13–16^, children with dyslexia, and cross-linguistically in ASD from this study, point to the possibility that RAN deficits may contribute to the language-related differences observed among these groups. Finally, evidence from studies of RAN in children also indicates clear stages of brain development in language-related regions related to RAN skills^79^, and shows that RAN naming time improves with developmental increases in myelin production and subsequent language development as well^80^. As such, together with findings from prior studies of RAN in ASD (and siblings and parents of individuals with ASD) in English-language samples, results from this study point towards such RAN differences as potential markers of ASD-related language impairments, with the possibility for revealing mechanistic underpinnings (specifically, refixations) of clinical phenotypes in ASD across different cultures and languages. It is also likely that findings in the present study were influenced by broader language, executive functioning, and processing speed differences not exclusive to ASD, particularly given evidence that RAN ability taps an extensive neural network reflecting a wide set of neuropsychological skills^3^. It is possible that the present findings documenting weaknesses in RAN in ASD cross-culturally are unlikely due to a deficit in one specific skill, but rather reflect atypicalities in a constellation of cognitive processes often functioning in concert as a result of the extensive neural network tapped by RAN^3^. Importantly, evidence that impairments in RAN performance (i.e., naming and error rates) are present across other language-related and neurodevelopmental conditions e.g., ^81^, highlights RAN’s utility for studying potentially shared neural and genetic mechanisms of impairment across conditions. This approach has been fruitfully explored in cross-population genetic studies, that have revealed many common genomic variations across disorders^82^. Future work would benefit from inclusion of an expanded battery of phenotypic correlates to further explore the clinical impact of RAN across cultures, and to disentangle language-related deficits associated with RAN^1,3,47,60,83^ from executive and visual-perceptual processes. Such fine-grained analyses will help to establish whether potential RAN deficits in ASD is language-related or not.

A consideration for future cross-linguistic studies of RAN is that naming in a non-native language for letter runs (1 of 4 conditions) in HK groups may have influenced the present study’s findings; however, this did not appear to impact ASD-related findings. In particular, naming time and refixations were the primary two indices revealing diagnostic effects only (i.e., without culture or interaction effects), suggesting that naming letters in English did not impact primary findings of diagnostic differences in refixations and naming time in both languages/cultures. Additionally, naming of letter runs occurred in a *separate* block or run, which ensured that only one language was being utilized within a specific block. Relatedly, non-symbolic naming may have been influenced by the stimuli included in these trials. Because Cantonese is a non-alphanumeric language and direct translations from English to Cantonese may cause initial phonemes to be similar, future work should consider the development and usage of items with unique initial-word sounds that may be important to determine if the diagnostic or cultural effects emerging from the current study in the non-symbolic conditions hold true. While prior work has indeed utilized Chinese characters for some RAN conditions^46,84,85^, these have not yet been normed, limiting application to cross-linguistic research studies and clinical settings. It may, however, be important to examine RAN performance in monolingual participants only, given known differences in bilingual studies of RAN^86^. However, as noted previously, findings reported here are not consistent with differences reported in studies of bilingual individuals, suggesting that bilingualism did not appear to impact current findings. Finally, it will be important to investigate whether differences in RAN extend to other Eastern Asian languages and cultures.

## Abbreviations

ADOS: Autism diagnostic observation schedule
ANOVA: Analysis of variance
AOI: Area of interest
ASD: Autism spectrum disorder
CTOPP: Comprehensive test of phonological processing
EVS: Eye-voice span
RAN: Rapid automatized naming
RRB: Restricted and repetitive behaviors
